# The limits of subfunctionalization

**DOI:** 10.1186/1471-2148-7-213

**Published:** 2007-11-07

**Authors:** Thomas MacCarthy, Aviv Bergman

**Affiliations:** 1 Department of Pathology, Albert Einstein College of Medicine, Bronx, NY 10461, USA; 2 Department of Neuroscience, Albert Einstein College of Medicine, Bronx, NY 10461, USA; 3 Department of Molecular Genetics, Albert Einstein College of Medicine, Bronx, NY 10461, USA

## Abstract

**Background:**

The duplication-degeneration-complementation (DDC) model has been proposed as an explanation for the unexpectedly high retention of duplicate genes. The hypothesis proposes that, following gene duplication, the two gene copies degenerate to perform complementary functions that jointly match that of the single ancestral gene, a process also known as subfunctionalization. We distinguish between subfunctionalization at the regulatory level and at the product level (e.g within temporal or spatial expression domains).

**Results:**

In contrast to what is expected under the DDC model, we use *in silico *modeling to show that regulatory subfunctionalization is expected to peak and then decrease significantly. At the same time, neofunctionalization (recruitment of novel interactions) increases monotonically, eventually affecting the regulatory elements of the majority of genes. Furthermore, since this process occurs under conditions of stabilizing selection, there is no need to invoke positive selection. At the product level, the frequency of subfunctionalization is no higher than would be expected by chance, a finding that was corroborated using yeast microarray time-course data. We also find that product subfunctionalization is not necessarily caused by regulatory subfunctionalization.

**Conclusion:**

Our results suggest a more complex picture of post-duplication evolution in which subfunctionalization plays only a partial role in conjunction with redundancy and neofunctionalization. We argue that this behavior is a consequence of the high evolutionary plasticity in gene networks.

## Background

**Figure 1:**
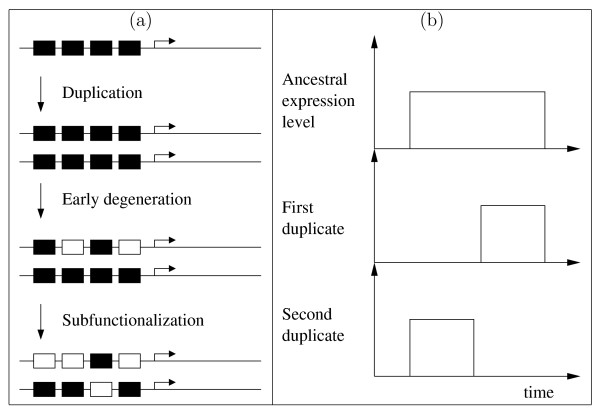
**The duplication-degeneration-complementation (DDC) model**. (a) A gene with four regulatory elements (black boxes), each controlling independent functions such as expression domains, is duplicated. Random null mutations in the regulatory elements (open boxes) through degeneration lead to subfunctionalization, where the regulatory elements complement each other to achieve the full ancestral repertoire. (b) Temporal subfunctionalization, illustrated here by the temporal expression patterns of a hypothetical ancestor and two evolved duplicates. The expression level of the duplicates has evolved such that the ancestral expression pattern is maintained in complementary temporal domains via the combined expression of the two duplicates.

The duplication-degeneration-complementation (DDC) model [1,2] has been proposed to explain the unexpectedly high retention of duplicate genes [3-5]. Briefly, the hypothesis proposes that, following gene duplication, redundant functions will degenerate at random from the daughter copies until their joint function matches that of the parent gene. The force for retention arises from the need to maintain ancestral functionality (and therefore requires only stabilizing selection). Originally proposed in the context of *cis*-regulatory elements, the model assumes regulatory elements with unique functions (e.g. spatial expression domains). In this context, each ancestral regulatory element is retained in at least one of the two daughter genes (Figure 1a). If each gene retains at least one ancestral element, while all redundant elements degenerate, we reach a state known as subfunctionalization. Perhaps the best studied example of this phenomenon involves the paralogous genes *Hoxa1 *and *Hoxb1 *in mouse development. *Hoxa1 *is highly sensitive to retinoic acid, and is important for segment identity in rhombomere 5 in the hindbrain. *Hoxb1 *is important for rhombomere 4 identity, and and is activated by *Hoxa1*, though its expression is maintained by autoregulation [6]. Tvrdik and Capecchi [7] reconstructed a hypothetical ancestral form of this system composed only of *Hoxa1 *with a *Hoxb1 *autoregulatory element introduced into its promoter region, and found no marked disparity with wild type. These results suggest that autoregulation and high retinoic acid sensitivity have degenerated in *Hoxa1 *and *Hoxb1 *respectively, leading to the current state of subfunctionalization. This example illustrates how spatial subfunctionalization (complementary expression in rhombomeres 4 and 5) is directly reflected by *cis*-regulatory subfunctionalization.

Subfunctionalization may also be observed in the temporal expression domain (Figure 1b). Following a gene duplication event, the temporal expression pattern of the ancestral singleton gene is maintained by the duplicate daughter genes. However, degeneration of the temporal expression pattern can lead to each duplicate being expressed in distinct, though complementary, time domains. Clearly, temporal subfunctionalization can occur independently, or together with, spatial subfunctionalization. Indeed, reported examples of temporal subfunctionalization [8-10] show both types coexisting.

Several authors have investigated the prevalence of subfunctionalization using genomic data. Here, it is often assumed that *cis*-regulatory binding motifs (or protein-DNA interactions found via chromatin immunoprecipitation) are equivalent to the independent regulatory elements of the DDC model. This approach was taken by Papp et al. [11], who examined the evidence for subfunctionalization in duplicated yeast genes and found that the number of shared regulatory motifs has decreased over time, while the total number of motifs has remained unchanged, suggesting an important additional role for neofunctionalization (i.e. the recruitment of novel interactions). Evangelisti and Wagner [12] reached similar conclusions. Adopting a similar approach, He and Zhang [13] analyzed both protein-protein interactions in yeast and spatial gene expression in human tissue, and suggested a new model termed subneofunctionalization. Under subneofunctionalization, gene duplication is followed by rapid subfunctionalization together with substantial and prolonged neofunctionalization.

Here, we adopt a modeling approach that integrates both network complexity and population-level dynamics to investigate the importance of subfunctionalization following gene duplication. The model is used to examine apparent discrepancies between existing genomic studies and the DDC model. In general terms, the relationship between subfunctionalization at the regulatory level (e.g. in *cis*-regulatory motifs) and at the level of the product (e.g. in temporal expression domains), remains unclear (with the exception of a some isolated examples, such as the mouse *Hox1 *genes mentioned above). This issue is also investigated using the model. The distinction we make between regulatory level and product level subfunctionalization should not be confused with genotype and phenotype respectively, since both levels (regulatory and product) involve genotypic (though not necessarily phenotypic) differences.

Broadly following the modeling framework of Siegal and Bergman [14], we consider a finite population of *M *individuals, each modeled as a gene regulatory network. It is assumed that the population has recently undergone a whole genome duplication, increasing the number of genes in each individual from *N *to 2*N*, while maintaining the same number (*N*) of protein products as before the duplication. The genes *i *and *i *+ *N *are paralogous. Each genotype is represented by a 2*N *× *N *interaction matrix *W*, the elements *W*_*i,j *_∈ {-1, 0, +1} represent the positive(+1), zero(0) or negative(-1) influence of product *j *(from genes *j *and *j *+ 1) on gene *i*.

At the network (phenotype) level, we adopt a Boolean model of gene regulation [15-17] which, though simple, captures essential features such as the threshold response [18], and additive regulation [19]. We do not assume each input can independently regulate its target, as in the DDC model, though such a scheme can indeed be represented. The phenotype corresponds to the temporal output s(*t*) of a dynamical system (see Methods – Network Dynamics) produced by the genotype (the matrix *W*), using initial conditions s(0) that are assigned randomly *a priori*, and are kept constant throughout each simulation.

All *M *individuals in the initial population are identical and are copies of a randomly generated founder individual. To create the founder, we generate a *N *× *N *matrix *Q*, with nonzero elements assigned at random with probability *c*_*i *_(the initial connectivity, or fraction of nonzero elements in the matrix *W*.). Each nonzero element is then assigned the value +1 or -1 with equal probability. The elements of *Q *are duplicated rowwise in the 2*N *× *N *founder matrix *W*, such that *W*_*i,j *_= *W*_*i*+*N,j *_= *Q*_*i,j*_. Subsequent generations are produced by cloning random individuals from the population (subject to mutation and selection). Here, the process is continued for 10000 generations.

Reproduction assumes mutation (in the form of changes to the cloned matrix *W*) at a rate *μ*. We make a distinction between a link deletion (where *W*_*i*,*j *_changes from -1 → 0, or +1 → 0) and a link addition (*W*_*i*,*j *_changes from 0 → -1, or 0 → +1, each with equal probability). Thus defined, mutation represents a broad range of mutation classes encompassing changes in *cis*-regulatory elements [20], alternative splicing regulation [21], or *trans*-acting factors [22] leading to link deletions or additions. Focusing on the regulatory level for mutations in this way is justified by recent work recognizing the overwhelming relative importance of regulatory divergence in paralog gene evolution [23]. We introduce a global deletion bias parameter *b *∈ (-1, 1), which defines a relative increase (if positive), or decrease (if negative) in probability for deletions (see Methods – Mutation). Deletion bias is chosen according to its observed effect on *c*_*f*_, the connectivity in the final generation (see Methods – Connectivity and deletion bias). The DDC model assumes that overall connectivity decreases as a consequence of the elimination of redundant interactions. The lower bound for connectivity under the hypothesis is *c*_*f *_= *c*_*i*_/2, since any further loss would start to eliminate non-redundant interactions. We therefore define the relative change in connectivity, *D *= *c*_*f*_/*c*_*i *_- 1, and examine two cases: *D *= 0 (*c*_*f *_= *c*_*i*_, no change in connectivity), and *D *= -0.5 (*c*_*f *_= *c*_*i*_/2, elimination of half the interactions, as expected under the DDC model).

We adopt a regime of strict stabilizing selection such that the phenotype, i.e. the temporal pattern of gene expression [**s**(0),...,**s**(*t*_*P*_)], remains identical through sucessive generations. This assumption concides with the neutrality premise of the DDC model, in that the combined behavior of each duplicated pair will be the same as that of the single ancestral gene. It also means there is no need to invoke positive selection. Unless otherwise stated, we assume *M *= 500, *N *= 10 and *μ *= 0.1 [14].

**Figure 2:**
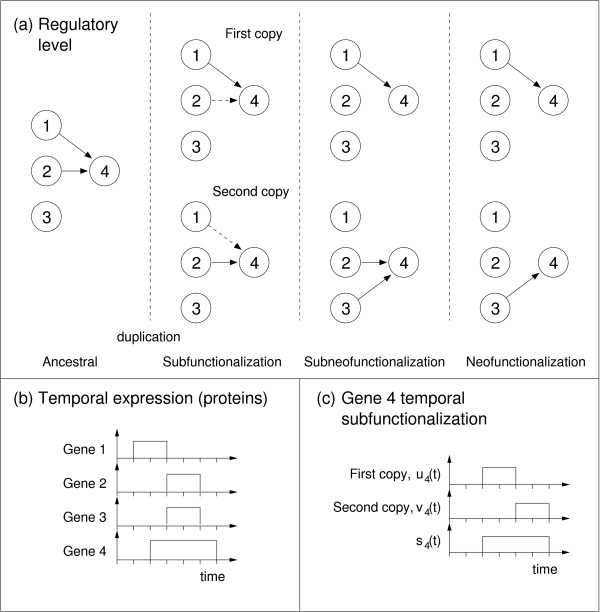
**A simple example of network evolution**. (a) At the regulatory level, gene 4 receives inputs from genes 1 and 2 in the ancestral state (inputs to genes 1, 2 and 3 not shown). A hypothetical protein expression pattern for this system is also shown (b). Following duplication and degeneration, regulatory subfunctionalization arises for gene 4 (dotted interactions are lost). A new input from gene 3 means we additionally have neofunctionalization, i.e., subneofunctionalization. After further degeneration (a, right) regulatory subfunctionalization is lost, while neofunctionalization is retained. (c) All three post-duplication states (sub-, subneo-, neo-functionalization) will result in temporal subfunctionalization for gene 4, since in the second and third timesteps only the first copy (*u*_4_) is ON, whereas in the fifth and sixth timesteps only the second copy (*v*_4_) is ON.

Throughout each simulation we measure connectivity, regulatory and temporal subfunctionalization and neofunctionalization (see Methods – Measures for paralogous genes). Briefly, regulatory subfunctionalization is considered to exist if some ancestral inputs (the inputs to gene *i *correspond to the *i*th row of matrix *W*) are lost in each of the paralogs, but together they still complement each other to represent the original input set. Neofunctionalization exists if new inputs evolve in either of the evolved paralogs. We also measure the number of shared links between two paralogs (*H*_*i*_, for ancestral gene *i*). Paralogs are considered to be temporally subfunctionalized (as the examples in figures 1 and 2 show) if one is ON and the other is OFF at a particular timepoint, and the reverse is true (OFF and ON respectively) at some other timepoint. We forsake spatial modeling and use only temporal modeling, focusing therefore on temporal subfunctionalization at the product level. Thus construed, the model allows us, for example, to relate regulatory changes (such as regulatory subfunctionalization and neofunctionalization) to product-level (in this case, temporal) subfunctionalization. Figure 2 shows a simple example of how regulatory changes (phenotypically neutral at the protein level) can lead through regulatory subfunctionalization, subneofunctionalization to neofunctionalization, while inducing temporal subfunctionalization at the product level.

Our *in silico *results show that, in contrast to what is expected under the DDC model, regulatory subfunctionalization peaks and then decreases significantly, while neofunctionalization increases monotonically, eventually affecting the majority of genes. These results are in agreement with existing bioinformatics studies [11-13]. We argue that this behavior is a consequence of the high evolutionary plasticity in gene networks [24,25]. Focusing on temporal subfunctionalization, we found it occurring at relatively modest frequencies, with the median not usually exceeding 20% of duplicate pairs across conditions. We compared these frequencies to a null ("unconstrained") model with no stabilizing selection (i.e. any mutation is accepted), giving us a distribution for the frequency of temporal subfunctionalization that would be expected by chance. From the comparison, we find that the actual frequencies observed are no higher than those of the null model, again contrary to expectations under the DDC model. We corroborated this finding using yeast microarray time-course data by showing that even the oldest paralogs exhibit similar frequencies of temporal subfunctionalization to what would be expected by chance. Lastly, using the model, we show that regulatory subfunctionalization does not necessarily cause subfunctionalization at the product level. We find that behavior analogous to genetic dominance in duplicate gene pairs creates the potential for escaping local minima in network space, thus dramatically simplifying network structure.

## Results and Discussion

### Regulatory subfunctionalization and neofunctionalization

**Figure 3:**
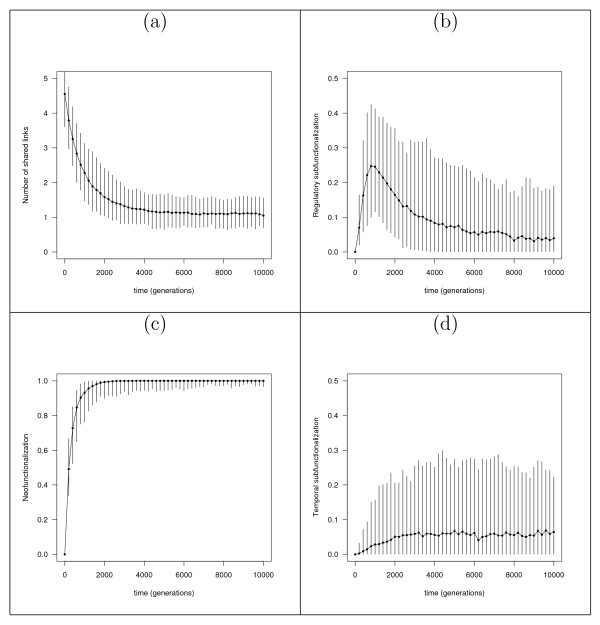
**Evolution of measures over time for a particular set of conditions (*c *= 0.45, *D *= 0)**. (a) Number of shared regulatory elements (*H*) between paralogous *cis*-regulatory elements declines over time. (b) Regulatory subfunctionalization (see Methods – Measures for paralogous genes) as a proportion of the number of eligible genes (defined as the number of rows with two or more nonzero entries), since we need to adjust for genes with *N *< 2, which cannot be subfunctionalized. (c) Neofunctionalization increases monotonically, then stabilizes. (d) Temporal subfunctionalization as a proportion of the number of genes. Graphs show median values and 95% confidence interval (errorbars) over 200 independent runs.

A previous study of cis-regulatory elements in yeast [11], has shown that, although the total number of regulatory motifs has remained unchanged over time (corresponding to relative change in connectivity, *D *= 0), the number of shared regulatory motifs in paralogous genes has decreased. Figure 3(a) shows how the model reproduces this behavior, observable in the progression of *H*_*i *_(the number of shared links between two paralogs, see Methods – Measures for paralogous genes) for a particular set of conditions (initial connectivity, *c*_*i *_= 0.45, *D *= 0). Across all conditions, we find that *H*_*i *_declines significantly (Mann-Whitney, *P *< 10^-16^, comparing initial to final generation for all cases, i.e. *c*_*i *_= 0.3, 0.45, 0.6 and *D *= 0, -0.5) in agreement with previous observations [11,12].

Clearly, if *H*_*i *_declines and connectivity remains the same (*D *= 0), then neofunctionalization must be playing an important role. Indeed, in all cases analyzed (including *D *= -0.5), median neofunctionalization increases monotonically, approaching a relatively high steady state value, as the example in figure 3(c) shows. We find that the most important factor determining final (steady state) neofunctionalization appears to be the deletion bias (Supp. Figure 2 in Additional file 1), with greater deletion bias leading to less neofunctionalization. This makes intuitive sense since, by definition, new links are less likely to be created when the deletion bias is higher.

**Figure 4:**
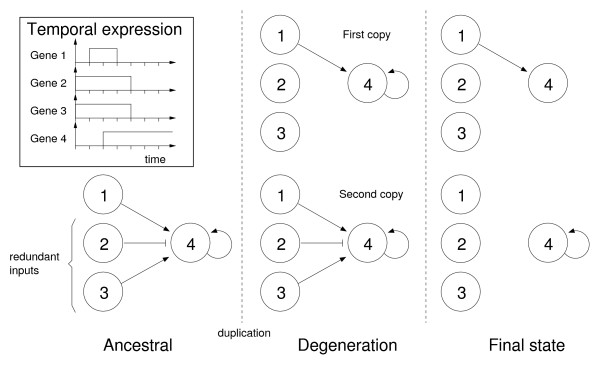
**An example of the dominance effect following duplication**. The inset (top left) gives an example time course for the protein products of the four genes. Regulation of gene 4 in the ancestral network includes two redundant interactions from genes 2 and 3, which cannot be removed in succession without perturbing the dynamics (since genes 2 and 3 have identical dynamics, their contributions cancel out). However, following duplication, these interactions can be lost successively (albeit in order, with the input from gene 3 degenerating first), since any dynamic perturbations will be masked by the intact second copy. Since the first copy now produces the correct dynamics, the degeneration process can be repeated in the second copy. Further degeneration might lead to a final state of complementation between the two copies.

Less intuitive is the progression of regulatory subfunctionalization. Figure 3(b) shows its progress over time for a particular set of conditions (though the results are qualitatively equivalent across all conditions). As predicted by the DDC model, the proportion of paralogous genes in a state of subfunctionalization increases following the genome duplication (at *t *= 0), as degeneration of redundant inputs occurs. Under the DDC model, we expect to observe a monotonical increase in subfunctionalization, reaching some stable peak. This should be particularly true of the case where mean connectivity declines (*D *= -0.5), since the theory predicts the degeneration of redundant links. However, in contradiction to the DDC model, the level of subfunctionalization peaks, and then falls to a final level significantly below this peak (Mann-Whitney comparing peak and final distributions, *P *< 10^-16 ^in all cases). Furthermore, we find that final subfunctionalization is reduced as we decrease *D *(Supp. Figure 3 in Additional file 1), an unexpected result since under the DDC model, we actually expect greater subfunctionalization as we decrease *D*. Note that under default conditions, we observe a certain amount of redundancy in the post-duplication network (see Methods – Connectivity and deletion bias). Generally speaking, a redundant interaction is any interaction that can be removed from the network with no phenotypic effect, i.e. without causing changes in the temporal expression pattern [**s**(0),...,**s**(*t*_*P*_)]; however, as the example in figure 4 shows, the phenotypic effect of removing a link may depend on how it is removed. If there are redundant interactions in the founder network, such interactions might be deleted in both duplicates during evolution with no phenotypic effect. In these circumstances, we would not recognize the gene as regulatory subfunctionalized according to the definition, in spite of the possibility that the remaining non-redundant interactions may in fact be subfunctionalized. In other words, if the founder network, prior to duplication, had consisted solely of non-redundant interactions, regulatory subfunctionalization would have been recognized. To minimize this unrecognized subfunctionalization, we repeated the simulations using *parsimonious *founder networks (see Methods – *Parsimonious *founder networks), in which no single interaction can be deleted without phenotypic change. We furthermore set *b *= 1 (i.e. only deletions will occur during evolution) to eliminate neofunctionalization. Even in these extreme conditions, we find a significant decline in regulatory subfunctionalization in two out of three cases (Mann-Whitney, *c*_*i *_= 0.3:*P *= 0.988, *c*_*i *_= 0.45: *P *= 3.7 × 10^-7^, *c*_*i *_= 0.6: *P *= 1.2 × 10^-5^). Since there is no neofunctionalization in this case, the result is somewhat surprising. A closer analysis shows however, that some *parsimonious *founder networks do indeed contain redundant interactions, albeit such that they can only be removed by two or more simultaneous deletions. After duplication, however, it may be possible for these interactions to degenerate in single steps due to a "dominance" effect, as shown in figure 4.

Although we enforce stabilizing selection at the level of the expression pattern [**s**(0),...,**s**(*t*_*P*_)], it could be argued that positive selection is not completely absent from the model, due to pressure on connectivity through the deletion bias parameter, *b*. This may be particularly true when *D *= -0.5, since here there is pressure to reduce the number of links in the network. However, we have just shown that the main results (the behavior over time of regulatory subfunctionalization and neofunctionalization) are qualitatively equivalent for both *D *= 0 and *D *= -0.5. It could further be argued that merely by having a nonzero deletion bias *b *(recall that *b *is chosen to obtain *D *= 0 or *D *= -0.5 as outcomes, see Methods -Connectivity and deletion bias) creates some degree of positive selection, since there is, by definition, a bias in choosing link deletions compared to link additions. However, in the particular case of *c*_*i *_= 0.6, both positive and negative values for deletion bias *b *were used (*b *was -0.173 and 0.497 for *D *= 0 and *D *= -0.5 respectively), suggesting that our main results also hold across positive and negative deletion bias values.

### Temporal subfunctionalization

Figure 3(d) shows an example for the progression of temporal subfunctionalization (see Methods – Measures for paralogous genes) with *c*_*i *_= 0.45. Across all conditions tested, we observe a relatively limited level of temporal subfunctionalization, not exceeding a median 8% of genes. In order to ensure these observations do not depend on the particular conditions used, it is informative to estimate an upper bound for temporal subfunctionalization. Intuitively, temporal subfunctionalization is most likely to occur when the founder network has minimal redundancy. Repeating these measurements using *parsimonious *founder networks, we do observe an increase relative to the non-*parsimonious *case, with temporal subfunctionalization stabilizing (at generation 10000) around a median value not exceeding 20%, although with very large variance. Even in this extreme case, the frequency of temporal subfunctionalization remains, in most cases, fairly limited. We also measured the prevalence of temporal subfunctionalization in the unconstrained model (see Supp. text and Supp. Figure 4 in Additional file 1), and, as expected, found an increase relative to the non-*parsimonious *case, with a median value again not exceeding 20%. Because there are no evolutionary constraints on the expression level of the paralogs in the unconstrained model, we can conclude from this result that temporal subfunctionalization in the "normal" (non-*parsimonious*) case, actually occurs at a lower frequency than would be expected by chance (Mann Whitney comparing final distributions, *P *< 10^-7 ^in all cases). Clearly, under the DDC hypothesis we would expect temporal subfunctionalization to be far more common.

**Figure 5:**
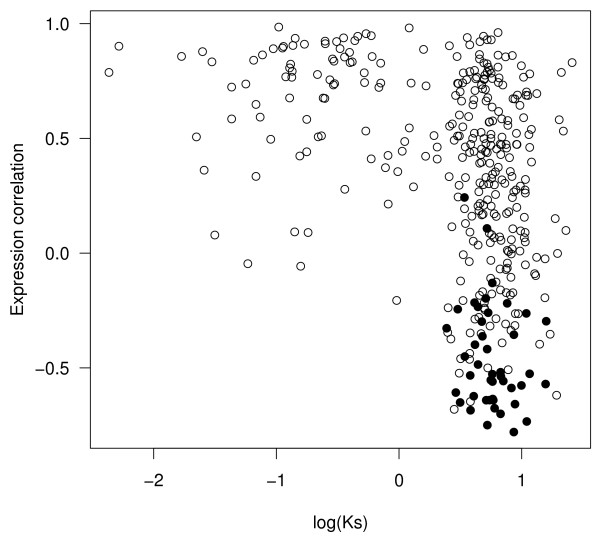
**Temporal subfunctionalization**. Observed temporal subfunctionalization *in vivo *for the "elutriation" time course dataset. *K*_*s *_values calculated for each yeast paralog (see Methods – Analysis of yeast data) are plotted against the correlation coefficient of the expression values. Paralogs for which temporal subfunctionalization is observed are shown with filled circles, those for which none is found are shown with open circles.

To corroborate our findings with biological data, we investigated the prevalence of temporal subfunctionalization *in vivo*. We proceeded by comparing paralogous genes in yeast (see Methods -Analysis of yeast data) to determine whether their expression (based on time-course data [26,27]) fit a pattern consistent with temporal subfunctionalization. Two paralogs are considered to be temporally subfunctionalized if one is ON and the other is OFF at a particular timepoint, and if the behaviour is reversed (OFF and ON respectively) at some other timepoint, within a single time-course (see Methods -Analysis of yeast data). Roughly speaking, we expect two expression patterns which are negatively correlated to exhibit a temporal subfunctionalization pattern with greater probability than if they were positively correlated. Figure 5 shows the correlation coefficient for each paralog pair in one time course ("elutriation") against the *K*_*s *_value, used here as a proxy for divergence time. Each point is labeled as temporally subfunctionalized (filled circles) or not (open circles).

Note that the thresholds used to decide OFF/ON states (in order to discretize the data) are arbitrary, such that varying the thresholds will change the observed proportion of temporal subfunctionalization. We therefore avoid directly comparing temporal subfunctionalization for the biological data with that for the simulated data, and compare them indirectly. We proceed by considering where the observed temporal subfunctionalization lies with respect to an appropriate null distribution using a permutation analysis. The null distribution was therefore obtained from random pairings (as opposed to paralog pairs) of time-courses by means of permutation. This null distribution was then used to estimate the probability (P-value) that actual temporal subfunctionalization is less than would be expected by chance. Table 1 shows the frequency of temporal subfunctionalization in youngest (lowest *K*_*s *_quartile) and oldest (highest *K*_*s *_quartile) groups, as well as the corresponding P-values. For the youngest (i.e. lowest) *K*_*s *_quartile, we find that actual temporal subfunctionalization was significantly less than would be expected by chance, whereas for the oldest *K*_*s *_quartile, the actual value was within the distribution. Most importantly, we see that, even for the oldest paralog pairs, actual temporal subfunctionalization is never greater than would be expected by chance, as was the case with the simulated data. This result contradicts the DDC model hypothesis.

**Table 1:** Temporal subfunctionalization frequencies

	Youngest		Oldest	
Dataset	f(TSF)	P	f(TSF)	P
alpha	0.015	0	0.108	0.075
cdc15	0.056	0.009	0.092	0.021
elutriation	0.011	0	0.130	0.068
*a*30	0.010	0	0.117	0.023
*a*38	0.010	0	0.150	0.205

Frequency of temporal subfunctionalization for the youngest (lowest *K*_*s *_quartile), and oldest (highest *K*_*s *_quartile) groups in each of the yeast time-course datasets. Also shown is the P-value for the randomized time-courses. Here, P < 0.025 indicates the actual frequency is significantly below that expected by chance (see Methods – Analysis of yeast data), and P > 0.975 that it is significantly greater.

### The relationship between regulatory and temporal subfunctionalization

**Figure 6:**
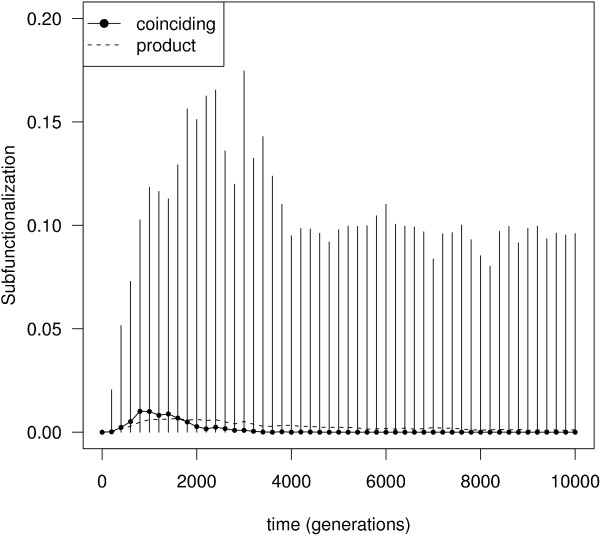
**Rates of coinciding regulatory and temporal subfunctionalization**. Rates of coinciding regulatory and temporal subfunctionalization under the same conditions as figure 3 (*c*_*i *_= 0.45, *D *= 0). The dotted line shows the product of the median independent frequencies. Graph shows median values and 95% confidence interval (errorbars) over 200 independent runs.

We observed above that regulatory and temporal subfunctionalization have different temporal patterns (compare Figure 3(b) and 3(d)). Regulatory subfunctionalization tends to peak rapidly followed by a prolonged decline, whereas temporal subfunctionalization tends to increase monotonically at a slower rate. From this observation, it appears unlikely that all temporal subfunctionalization will be caused by regulatory subfunctionalization in normal conditions. We measured the frequency of genes with coinciding regulatory and temporal subfunctionalization (i.e. the fraction of *N *× *M *paralog pairs having both regulatory and temporal subfunctionalization simultaneously). If the two types of subfunctionalization are independent of one another, we expect the coinciding frequency to equal the product of their independent frequencies. whereas if regulatory subfunctionalization causes temporal subfunctionalization, the coinciding frequency should be higher. Figure 6 shows the two frequencies, under the same conditions as figure 3 (*c*_*i *_= 0.45, *D *= 0), are very close. Across all conditions, the coinciding frequency was not found to be significantly greater than the product of independent frequencies (Mann-Whitney comparing final distributions, *P *> 0.999 in all cases).

Repeating this procedure using *parsimonious *founder networks and *b *= 1 as before, should allow us to obtain an upper bound for coinciding regulatory and temporal subfunctionalization. Under these extreme conditions, we do indeed observe that the coinciding frequency is significantly greater than the product (Mann-Whitney, *P *< 1.7 × 10^-6 ^for *c*_*i *_= 0.3, 0.45, 0.6). Interestingly though, even in this case, we find that temporal subfunctionalization is not always due to regulatory subfunctionalization (although the variance across simulations is very large). In these conditions, it may be hard to imagine how temporal subfunctionalization can occur without regulatory subfunctionalization. As before, we find that redundant interactions, albeit such that they can only be removed by two or more simultaneous deletions, explain this phenomenon (see Figure 4).

## Conclusion

Our results confirm previous analyses revealing the coexistence of subfunctionalization and neofunctionalization in biological networks following gene duplication [11-13]. This was unexpected since we have adopted a stabilizing selection model that is expected to favor subfunctionalization alone, according to the assumptions of the DDC model. In particular, it is unnecessary to invoke positive selection to explain the high prevalence of neofunctionalization. Our results can be explained in terms of evolution in neutral spaces [28]. Previous studies of gene networks [24,25] and RNA folding [29] have illustrated the prevalence in biological systems of evolutionary plasticity in combination with phenotypic neutrality [30]. We have shown how this evolutionary plasticity enables the coexistence of subfunctionalization and neofunctionalization, as has been observed in genomic studies.

The differences between our model and the DDC model arise from the distinct scope of each model. The scope for the DDC model is the individual (duplicated) pair of genes, in which inputs are assumed to represent nonredundant functions. However, a network composed entirely of such genes would lack robustness (any link deletion would, at a minimum, change the output of the target gene), which contradicts available evidence [14,31,32]. In contrast, in our model the phenotype is a consequence of the behaviour of the entire network. Here, given adequate conditions (deletion bias, *b *< 1), we expect a certain amount of redundancy, and therefore robustness, to exist. Indeed, such redundancy is expected as an outcome of the evolutionary process [33]. A recent study of divergent regulatory architectures associated with mating type in yeast [34] inferred a succession of phenotypically neutral changes in which an ancestral transcription activator (present in *C. albicans*) was replaced by a repressor in modern *S. cerevisiae*, via an ancestor in which both activator and repressor were simultaneously present, illustrating the importance of redundancy in evolution. Other studies have shown a widespread turnover of transcription factor binding sites in both mammals [35] and insects [36], and occurring even under conditions of stabilizing selection [37].

Clearly, in our model, if two or more genes have the same expression pattern, **s**(*t*), over time, then target genes can switch between these inputs with no phenotypic consequences. Such a switch is likely to occur via an intermediate genotype in which both inputs are simultaneously present. From the earliest analyses of time-course data from DNA microarrays [38], it has been clear that many genes share very similar temporal expression patterns [39], a fact that would facilitate switching of the type described. Recent evidence from the segmentation clock (the oscillatory network controlling vertebrate somite development) suggests that current models based on a small number of elements [40,41] need to be revised in the light of findings implicating a large network of inter-related components [42], all of which are regulated periodically. Although we should exercise caution in comparing model gene networks with the segmentation clock (which additionally involves cell-signaling and dynamic complex formation), it seems likely that the presence of a greater number of periodically-expressed genes increases the opportunities for interaction turnover. Recent studies have observed significant divergence of gene expression between paralogous genes [43,44]. Even under the neutrality conditions of our model, any variation is tolerated so long as the expression dynamics are unaffected. This is a consequence of the threshold response of each gene, which is dependent only on the sign (not the magnitude) of the combined inputs. Thus, the fact that the threshold response is a key feature of gene regulation [18,45] suggests an explanation for gene expression variation in both our model and observed data.

Our model assumes a whole genome duplication (WGD) event. Such events have made major contributions of duplicated genes in vertebrates [46], plants [47-49] and yeast [50]. However, we want to emphasize that our conclusions also extend to smaller scale duplications, including single gene duplications. Note that, in the model, the output of each gene is unaffected by duplication. Because the network outputs (and therefore the inputs also) remain identical throughout the simulation, each paralog pair evolves independently, irrespective of whether the other genes are duplicated or not. Therefore, even if only a subset of genes are duplicated, this subset would evolve in the same way as it would following WGD. Clearly though, this argument applies only to duplication events which are phenotypically neutral (an assumption of our model). Many small-scale duplication events involving, for example, proteins that are active as protein complexes, may be deleterious due to dosage effects [51].

We also needed to verify that the results using the yeast data apply to duplicates not originating from the WGD. For this purpose, we used a published list of gene pairs formed by WGD [52], and removed these from our original dataset. To ensure we were using gene pairs in a comparable age range, we also removed those paralogs with *K*_*s *_values outside the range observed for the WGD dataset. We then repeated the analysis, and found the frequency of temporal subfunctionalization to be not significantly higher than would be expected by chance, as with the original dataset (alpha: *P *= 0.059, cdc15: *P *= 0.004, elutriation: *P *= 0.031, *a*30: *P *= 0.001, *a*38: *P *= 0.004).

Notwithstanding its convenience in terms of data availability, yeast is not optimal for studying subfunctionalization due to its large effective population size, *M*. Following duplication, neutral (possibly subfunctionalized) alleles take in the order of *M *generations to reach fixation, thus we would expect the incidence of subfunctionalization to be lower when *M *is large. Although this effect is somewhat attenuated by the use of laboratory yeast strains (that have likely been subjected to periodic bottlenecks), our yeast data analysis, as well as the results of previous genomic studies [11-13], should be interpreted with certain caution for this reason. Our theoretical results, on the other hand, use a relatively small population size (*M *= 500), in which one would expect higher subfunctionalization. In spite of the different population sizes, the similarities between our theoretical results and those of genomic analyses (using yeast), suggest that the overall pattern of subfunctionalization and neofunctionalization evolution following duplication is similar. As suitable data becomes available for a wider range of organisms, it will become possible to evaluate more effectively the effects of population size in this context.

Although it would be fair to say that the model of gene regulation we have chosen is somewhat crude, our choice has been deliberate. Our model captures essential features of gene network behavior (e.g. threshold response) and emphasizes the importance of transcription regulation in evolution [20,53,54] resulting in neutral space properties that apply to real gene regulatory networks [28]. We consider that choosing a more sophisticated model would have resulted in qualitatively equivalent results, but with reduced explanatory power. An important outcome of this investigation has been to show the substantial benefits that arise from considering the behavior of the entire network as a system, as opposed to considering the individual genes in isolation. Our results suggest that subfunctionalization alone cannot explain the high retention of duplicate genes. At the same time, a more complex picture of post-duplication evolution emerges in which redundancy and neofunctionalization play important roles alongside subfunctionalization.

## Methods

### Network dynamics

Network behavior is determined (using the 2*N *× *N *interaction matrix *W*) by a Boolean dynamical system of the form *s*_*i*_(*t *+ 1) = *σ*(*u*_*i*_(*t*) + *v*_*i*_(*t*)) for the *i*th protein product, where

ui(t)=σ(∑jNWi,jsj(t))
 MathType@MTEF@5@5@+=feaafiart1ev1aaatCvAUfKttLearuWrP9MDH5MBPbIqV92AaeXatLxBI9gBaebbnrfifHhDYfgasaacPC6xNi=xI8qiVKYPFjYdHaVhbbf9v8qqaqFr0xc9vqFj0dXdbba91qpepeI8k8fiI+fsY=rqGqVepae9pg0db9vqaiVgFr0xfr=xfr=xc9adbaqaaeGacaGaaiaabeqaaeqabiWaaaGcbaGaemyDau3aaSbaaSqaaiabdMgaPbqabaGccqGGOaakcqWG0baDcqGGPaqkcqGH9aqpiiGacqWFdpWCcqGGOaakdaaeWbqaaiabdEfaxnaaBaaaleaacqWGPbqAcqGGSaalcqWGQbGAaeqaaOGaem4Cam3aaSbaaSqaaiabdQgaQbqabaGccqGGOaakcqWG0baDcqGGPaqkcqGGPaqkaSqaaiabdQgaQbqaaiabd6eaobqdcqGHris5aaaa@46B8@

vi(t)=σ(∑jNWi+N,jsj(t))
 MathType@MTEF@5@5@+=feaafiart1ev1aaatCvAUfKttLearuWrP9MDH5MBPbIqV92AaeXatLxBI9gBaebbnrfifHhDYfgasaacPC6xNi=xI8qiVKYPFjYdHaVhbbf9v8qqaqFr0xc9vqFj0dXdbba91qpepeI8k8fiI+fsY=rqGqVepae9pg0db9vqaiVgFr0xfr=xfr=xc9adbaqaaeGacaGaaiaabeqaaeqabiWaaaGcbaGaemODay3aaSbaaSqaaiabdMgaPbqabaGccqGGOaakcqWG0baDcqGGPaqkcqGH9aqpiiGacqWFdpWCcqGGOaakdaaeWbqaaiabdEfaxnaaBaaaleaacqWGPbqAcqGHRaWkcqWGobGtcqGGSaalcqWGQbGAaeqaaOGaem4Cam3aaSbaaSqaaiabdQgaQbqabaGccqGGOaakcqWG0baDcqGGPaqkcqGGPaqkaSqaaiabdQgaQbqaaiabd6eaobqdcqGHris5aaaa@48C1@

and

σ(x)={1if x>00otherwise
 MathType@MTEF@5@5@+=feaafiart1ev1aaatCvAUfKttLearuWrP9MDH5MBPbIqV92AaeXatLxBI9gBaebbnrfifHhDYfgasaacPC6xNi=xI8qiVKYPFjYdHaVhbbf9v8qqaqFr0xc9vqFj0dXdbba91qpepeI8k8fiI+fsY=rqGqVepae9pg0db9vqaiVgFr0xfr=xfr=xc9adbaqaaeGacaGaaiaabeqaaeqabiWaaaGcbaacciGae83WdmNaeiikaGIaemiEaGNaeiykaKIaeyypa0ZaaiqaaeaafaqaaeGacaaabaGaeGymaedabaacbaGae4xAaKMae4NzayMaeeiiaaIaemiEaGNaeyOpa4JaeGimaadabaGaeGimaadabaGae43Ba8Mae4hDaqNae4hAaGMae4xzauMae4NCaiNae43DaCNae4xAaKMae43CamNae4xzaugaaaGaay5Eaaaaaa@4860@

Starting from an initial state vector **s**(0) ∈ {0, 1}^*N*^, successive states **s**(*t*) are generated until we encounter a repeated state **s**(*t*_*P*_) (*t*_*P *_> 0), such that **s**(*t*_*P*_) = **s**(*t*_*R*_) for some *t*_*R *_<*t*_*P *_. The initial state **s**(0) is constant for each simulation and is set by randomly choosing each *s*_*i *_= 0 or 1.

### Mutation

The probabilities for deletion and addition of a nonzero element (*W*_*i*,*j*_) of the interaction matrix *W*, are

pd=m(1+b)k
 MathType@MTEF@5@5@+=feaafiart1ev1aaatCvAUfKttLearuWrP9MDH5MBPbIqV92AaeXatLxBI9gBaebbnrfifHhDYfgasaacPC6xNi=xI8qiVKYPFjYdHaVhbbf9v8qqaqFr0xc9vqFj0dXdbba91qpepeI8k8fiI+fsY=rqGqVepae9pg0db9vqaiVgFr0xfr=xfr=xc9adbaqaaeGacaGaaiaabeqaaeqabiWaaaGcbaGaemiCaa3aaSbaaSqaaiabdsgaKbqabaGccqGH9aqpcqWGTbqBjuaGdaWcaaqaaiabcIcaOiabigdaXiabgUcaRiabdkgaIjabcMcaPaqaaiabdUgaRbaaaaa@3848@

and

pa=m(1−b)k
 MathType@MTEF@5@5@+=feaafiart1ev1aaatCvAUfKttLearuWrP9MDH5MBPbIqV92AaeXatLxBI9gBaebbnrfifHhDYfgasaacPC6xNi=xI8qiVKYPFjYdHaVhbbf9v8qqaqFr0xc9vqFj0dXdbba91qpepeI8k8fiI+fsY=rqGqVepae9pg0db9vqaiVgFr0xfr=xfr=xc9adbaqaaeGacaGaaiaabeqaaeqabiWaaaGcbaGaemiCaa3aaSbaaSqaaiabdggaHbqabaGccqGH9aqpcqWGTbqBjuaGdaWcaaqaaiabcIcaOiabigdaXiabgkHiTiabdkgaIjabcMcaPaqaaiabdUgaRbaaaaa@384D@

respectively, where m=μ2N2
 MathType@MTEF@5@5@+=feaafiart1ev1aaatCvAUfKttLearuWrP9MDH5MBPbIqV92AaeXatLxBI9gBaebbnrfifHhDYfgasaacPC6xNi=xH8viVGI8Gi=hEeeu0xXdbba9frFj0xb9qqpG0dXdb9aspeI8k8fiI+fsY=rqGqVepae9pg0db9vqaiVgFr0xfr=xfr=xc9adbaqaaeGacaGaaiaabeqaaeqabiWaaaGcbaGaemyBa0Maeyypa0tcfa4aaSaaaeaaiiGacqWF8oqBaeaacqaIYaGmcqWGobGtdaahaaqabeaacqaIYaGmaaaaaaaa@33C2@ is the mutation rate per element, *b *is a global deletion bias parameter *b *∈ (-1, 1), and *k *is a normalizing factor to ensure that the mutation rate per genotype (*μ*) remains constant. To find *k*, we proceed as follows. In a matrix with connectivity *c*, the probability of a deletion is *cpd*, and the probability of an addition is (1 - *c*)*p*_*a*_. To maintain a mutation rate of *m *per element, we require

cpd+(1−c)pa=μ2N2=m
 MathType@MTEF@5@5@+=feaafiart1ev1aaatCvAUfKttLearuWrP9MDH5MBPbIqV92AaeXatLxBI9gBaebbnrfifHhDYfgasaacPC6xNi=xI8qiVKYPFjYdHaVhbbf9v8qqaqFr0xc9vqFj0dXdbba91qpepeI8k8fiI+fsY=rqGqVepae9pg0db9vqaiVgFr0xfr=xfr=xc9adbaqaaeGacaGaaiaabeqaaeqabiWaaaGcbaGaem4yamMaemiCaa3aaSbaaSqaaiabdsgaKbqabaGccqGHRaWkcqGGOaakcqaIXaqmcqGHsislcqWGJbWycqGGPaqkcqWGWbaCdaWgaaWcbaGaemyyaegabeaakiabg2da9maalaaabaacciGae8hVd0gabaGaeGOmaiJaemOta40aaWbaaSqabeaacqaIYaGmaaaaaOGaeyypa0JaemyBa0gaaa@4186@

Substituting, we obtain

m[c(1+b)k+(1−c)(1−b)k]
 MathType@MTEF@5@5@+=feaafiart1ev1aaatCvAUfKttLearuWrP9MDH5MBPbIqV92AaeXatLxBI9gBaebbnrfifHhDYfgasaacPC6xNi=xI8qiVKYPFjYdHaVhbbf9v8qqaqFr0xc9vqFj0dXdbba91qpepeI8k8fiI+fsY=rqGqVepae9pg0db9vqaiVgFr0xfr=xfr=xc9adbaqaaeGacaGaaiaabeqaaeqabiWaaaGcbaGaemyBa02aamWaaeaacqWGJbWyjuaGdaWcaaqaaiabcIcaOiabigdaXiabgUcaRiabdkgaIjabcMcaPaqaaiabdUgaRbaakiabgUcaRiabcIcaOiabigdaXiabgkHiTiabdogaJjabcMcaPKqbaoaalaaabaGaeiikaGIaeGymaeJaeyOeI0IaemOyaiMaeiykaKcabaGaem4AaSgaaaGccaGLBbGaayzxaaaaaa@4440@

The term within parentheses must be equal to 1, and therefore

*k *= *c*(1+*b*) + (1-*c*)(1-*b*)

Note that if the deletion bias is set to its highest value *b *= 1, then *p*_*a *_= 0 and only link deletions occur. Similarly, if it is set to its lowest value *b *= -1, then *p*_*d *_= 0 and only link additions occur.

### Connectivity and deletion bias

It is convenient to elucidate the effect deletion bias (*b*) has on connectivity (*c*) as the population evolves, and in particular, the effect of *b *on *c*_*f*_, the final value of *c *at generation 10000. Intuitively, one would expect high *b *values (*b*~1) to reduce connectivity, when compared to lower *b *values (*b*~-1). Simulations were performed across a range of values for *b *(*b *= -1, -0.5, 0, 0.5, 1), initial connectivity (*c*_*i *_= 0.3, 0.45, 0.6). In all cases we find the relationship between *b *and median *c*_*f *_to be approximately linear. We also find that, irrespective of *c*_*i*_, a large range for *c*_*f *_is possible. Even for relatively high initial connectivity *c*_*i *_= 0.6 (Supp. Figure 1 in Additional file 1, right) connectivity can be reduced to well below half the initial value (compare *c*_*i*_/2 = 0.3 with 0.215, the upper bound for 95% confidence interval), a decline beyond that predicted by the DDC model. Note that the possibility of reducing *c*_*f *_to below *c*_*i*_/2 suggests that there is redundancy in the founder network, before duplication.

We define the relative change in connectivity, *D *= *c*_*f*_/*c*_*i *_- 1. Under the DDC model, we expect a long-term decline in connectivity to *D *= -0.5. We examine the two extremes: *D *= 0 (no change in connectivity), and *D *= -0.5 (elimination of half the interactions). Again a range of conditions are investigated for initial connectivity (*c*_*i *_= 0.3, 0.45, 0.6). In all cases, the appropriate deletion bias (*b*) is estimated using linear regression results from the relevant dataset: for example, a simulation with initial connectivity, *c*_*i *_= 0.3 (Supp. Figure 1 in Additional file 1, left) requires a value of *b *≃ 0.43 to attain *D *= 0 (i.e. *c*_*f *_= 0.3).

### Measures for paralogous genes

Recall that, in the initial population, all genotypes are identical copies of a 2*N *× *N *matrix *W*, and that this matrix is generated by rowwise duplication of a random *N *× *N *matrix *Q*, such that *W*_*i,j *_= *W*_*i*+*N*,*j *_= *Q*_*i,j*_. We measure regulatory subfunctionalization by comparing paralogous genes in some evolved genotype, by comparing the rows *W*_*i *_and *W*_*i*+*N*_, with the ancestral row *Q*_*i*_. We define a simple qualitative measure to detect subfunctionalization. We define *F*_*i *_as the set of indices *j*_±_, such that *Q*_*ij *_≠ 0, representing the original inputs to gene *i*, and distinguishing between positive (*j*_+_, Qij+
 MathType@MTEF@5@5@+=feaafiart1ev1aaatCvAUfKttLearuWrP9MDH5MBPbIqV92AaeXatLxBI9gBaebbnrfifHhDYfgasaacPC6xNi=xH8viVGI8Gi=hEeeu0xXdbba9frFj0xb9qqpG0dXdb9aspeI8k8fiI+fsY=rqGqVepae9pg0db9vqaiVgFr0xfr=xfr=xc9adbaqaaeGacaGaaiaabeqaaeqabiWaaaGcbaGaemyuae1aaSbaaSqaaiabdMgaPjabdQgaQnaaBaaameaacqGHRaWkaeqaaaWcbeaaaaa@30FC@ = +1) and negative (*j*_-_, Qij−
 MathType@MTEF@5@5@+=feaafiart1ev1aaatCvAUfKttLearuWrP9MDH5MBPbIqV92AaeXatLxBI9gBaebbnrfifHhDYfgasaacPC6xNi=xH8viVGI8Gi=hEeeu0xXdbba9frFj0xb9qqpG0dXdb9aspeI8k8fiI+fsY=rqGqVepae9pg0db9vqaiVgFr0xfr=xfr=xc9adbaqaaeGacaGaaiaabeqaaeqabiWaaaGcbaGaemyuae1aaSbaaSqaaiabdMgaPjabdQgaQnaaBaaameaacqGHsislaeqaaaWcbeaaaaa@3107@ = -1) inputs. We define similar sets *A*_*i*_, *B*_*i *_for the rows *W*_*i *_and *W*_*i*+*N *_in the evolved genotype, representing the inputs to the paralogous genes. Subfunctionalization exists if some original inputs have been lost in each of the paralogs, but together they still complement each other to represent the original input set, i.e., if the following three conditions are met:

|*F*_*i*_| > |*A*_*i *_∩ *F*_*i*_| > 0

|*F*_*i*_| > |*B*_*i *_∩ *F*_*i*_| > 0

(*A*_*i *_∪ *B*_*i*_) ∩ *F*_*i *_= *F*_*i*_

Note that we need |*F*_*i*_| ≥ 2 for regulatory subfunctionalization to be possible.

Neofunctionalization exists if there are any new inputs in either of the evolved paralogs, i.e.

|(*A*_*i *_∪ *B*_*i*_) - *F*_*i*_| > 0

We define the number of shared links between two paralogs, as *H*_*i *_= |*A*_*i *_∩ *B*_*i*_|.

To measure temporal subfunctionalization, we consider paralogs as subfunctionalized if one is ON and the other is OFF at a particular timepoint, and if the behaviour is reversed (OFF and ON respectively) at some other timepoint. More formally, if we define time courses for the two paralogs as *u*_*i*_(*t*) and *v*_*i*_(*t*) (as above, under "Network dynamics"), then the conditions are *u*_*i*_(*t*_*X*_) = 1, *v*_*i*_(*t*_*X*_) = 0, *u*_*i*_(*t*_*Y*_) = 0, *v*_*i*_(*t*_*Y*_) = 1. *t*_*X *_≠ *t*_*Y *_.

### *Parsimonious *founder networks

If there are redundant interactions in the founder network, such interactions can be deleted in both duplicates during evolution with no phenotypic effect. Consequently, regulatory subfunctionalization would not be recognized, in spite of the possibility that the remaining non-redundant interactions may in fact be subfunctionalized. To address this issue, we generate *parsimonious *(i.e. with minimal redundancy) founder networks. We implement the following algorithm to obtain networks with (approximate) initial connectivity *c*_*i*_:

1. Generate a matrix *Q' *with full connectivity (*c *= 1), and generate **s**(*t*).

2. Delete connections in random order, retaining only those deletions which do not alter the expression pattern, **s**(*t*).

3. Repeat step 2 until all attempted deletions are unsuccessful, i.e. alter **s**(*t*).

4. Accept *Q' *as new founder *Q *if it has connectivity (*c*) between *c*_i _- Δ_*p *_and *c*_*i *_+ Δ_*p*_, otherwise return to step 1 (Δ_*p *_= 0.05 was used).

The matrix *Q *is then duplicated to create the matrix *W *in the initial population. The algorithm works because, for a large sample of initial random matrices *Q'*, one observes *c *values (for the founder matrices *Q*) across the entire range [0, 1].

### Analysis of yeast data

We used the program *GenomeHistory *[55] with the same parameters as used for *Saccharomyces cerevisiae *in the original study, resulting in a list of paralogous genes. The program also estimates the number of synonymous (*K*_*s*_) substitutions per synonymous site, and the number of nonsynonymous (*K*_*a*_) substitutions per nonsynonymous site. Following Evangelisti and Wagner [12] we retained only gene pairs with *K*_*a *_< 1 for further analysis. We use the *K*_*s *_value as a proxy for divergence time. Because we make only broad categorizations based on *K*_*s*_, we have retained the lower accuracy *K*_*s *_values labeled as "saturated" by *GenomeHistory*. Cell-cycle synchronized microarray data for yeast was obtained from two sources: three distinct time-courses (labeled as "alpha","cdc15", and "elutriation") were obtained from the first [26], and two time-courses (labeled as "*α*30" and "*α*38") from a second, more recent, dataset [27] (the "cdc28" time-course from the first dataset was excluded due to its containing many missing values, and the lower-resolution "*α*26" time-course was excluded from the second dataset).

Paralogs *A *and *B *are considered to be temporally subfunctionalized if A is ON and B is OFF at some time *t*_*X *_and A is OFF and B is ON at some other time *t*_*Y*_. Since the data are continuous, these need to be discretized beforehand. Each gene and time-course [time series *S*(*t*)] were discretized independently by normalizing *S*(*t*) to the interval (0, 1) to give a series *S'*(*t*), then assigning ON values where *S'*(*t*) > *θ*, and OFF values where *S'*(*t*) < 1 - *θ*. To verify that true temporal subfunctionalization has occurred, we used subcellular localization data [56] to exclude paralogs that do not co-localize.

The null distribution (representing the distribution of temporal subfunctionalization that would be expected by chance) was generated by taking the paralogous pairs and randomly shuffing the partners, for example in a dataset with 3 paralogous pairs, (*x*_1_, *y*_1_),(*x*_2_, *y*_2_), (*x*_3_, *y*_3_) → (*x*_1_, *y*_3_), (*x*_2_, *y*_1_), (*x*_3_, *y*_2_). Separate datasets were generated for the "youngest" (i.e. lowest *K*_*s*_) and "oldest" (i.e. highest *K*_*s*_) quartiles for *K*_*s *_in each time-course. Temporal subfunctionalization was then measured for 1000 random shuffes. These measurements were then used to estimate the probability (P-value) that actual temporal subfunctionalization is less than would be expected by chance, defined as the fraction of random shuffes for which temporal subfunctionalization is below the actual value. All results shown use *θ *= 0.8. However, we repeated the analysis using *θ *through the range (0.5, 0.9), and obtained qualitatively equivalent results, as shown in Supp. Table 1 in Additional file 1.

## Authors' contributions

Both TM and AB conceived the project, executed experiments and prepared the manuscript.

## Supplementary Material

Additional file 1 Supporting text, figures and table. Supplementary text: Analysis of unconstrained model. Supplementary figures 1 to 4, and Supplementary table 1. Click here for file
