# Supplementary material for: The limits of subfunctionalization
Source: BMC Evol Biol. 2007 Nov 7;7:213. doi: 10.1186/1471-2148-7-213 (PMC2213666; doi:10.1186/1471-2148-7-213)
Supplement: Additional file 1 — Supporting text, figures and table. Supplementary text: Analysis of unconstrained model. Supplementary figures 1 to 4, and Supplementary table 1. [file 1471-2148-7-213-S1.pdf]

# Supplementary text, figures and table for "The limits of subfunctionalization"

## Supplementary text: Analysis of unconstrained model

Here we consider a simpler unconstrained model in which the population evolves free from the constraint for dynamic neutrality, i.e. all mutations are accepted, even if they change the expression pattern, $s(t)$. In this instance, we can derive analytically the approximate relationship between evolved connectivity and deletion bias.

The probability that a particular matrix element is nonzero at time $t$ is $P_t$, the initial probability is $c_i = P_0$, and the mutation rate per element is $m$. After one timestep ($t = 1$), the probability the element has been deleted, i.e. changed from nonzero to zero, is

$$c_i m \frac{(1+b)}{k} = P_0 m \frac{(1+b)}{k}$$

whereas the probability the element has been added, i.e. changed to nonzero from zero is

$$(1-c_i) m \frac{(1-b)}{k} = (1-P_0) m \frac{(1-b)}{k}$$

Combining these two terms, we have

$$P_1 = P_0 - P_0 m \frac{(1+b)}{k} + (1-P_0) m \frac{(1-b)}{k}$$

More generally,

$$P_{t+1} = P_t - P_t m \frac{(1+b)}{k} + (1-P_t) m \frac{(1-b)}{k}$$

At steady state, $P_{t+1}$ and $P_t$ are equal

$$P_{t+1} - P_t = 0 = P_t - P_t m \frac{(1+b)}{k} + (1-P_t) m \frac{(1-b)}{k} - P_t$$

$$P_t(1+b) = (1-P_t)(1-b)$$

$$P_t = c_f = \frac{1-b}{2}$$

Note that the final connectivity $c_f$ of the matrix is the same as the steady state probability $P_t$ of having a nonzero matrix element. There is therefore, a simple linear relationship final connectivity in this model depends on a simple linear relationship with the deletion bias $b$. These results are intuitively obvious: if $b = 1$ (only deletions are applied), then all elements will be eliminated and final connectivity, $c_f = 0$. Similarly, if $b = -1$ (only additions are applied), then $c_f = 1$.

We will also be interested in knowing the deletion bias $b$ necessary to obtain a particular change in connectivity, $D = c_f/c_i - 1$.

$$D = \frac{1-b}{2c_i} - 1$$

$$b = 1 - 2c_i(1 + D)$$

Although this relationship is informative (it will be used to assign $b$), we are mainly interested how subfunctionalization, both regulatory and temporal, and neofunctionalization behave for the unconstrained model. Simulations using the unconstrained model show that this behaviour is qualitatively equivalent to that of the constrained model in similar conditions, as shown in Supp. Figure 4.

# Supplementary figures

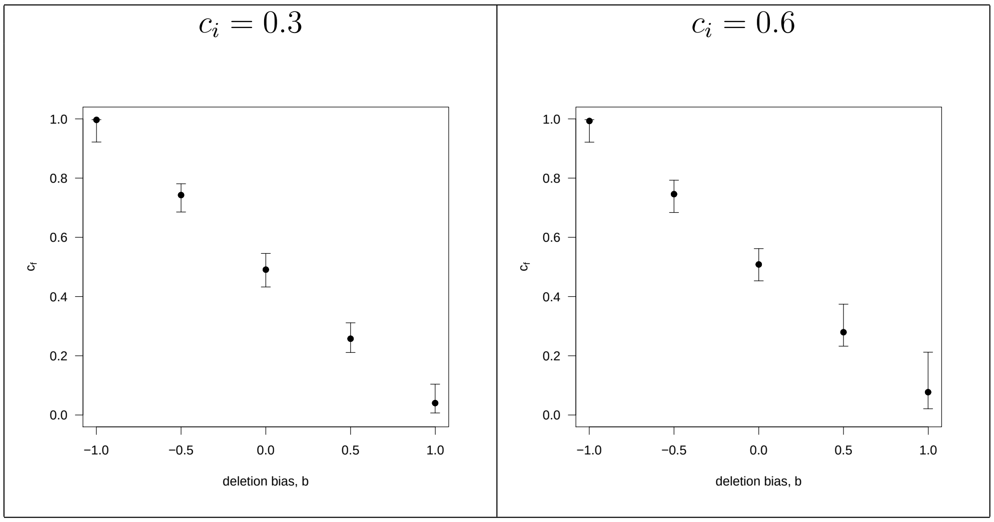


**Supplementary Figure 1.** The effect of deletion bias, $b$, on final connectivity, $c_f$, in the model. Shown are graphs for two initial connectivity levels: (a) $c$=0.3 and (b) $c$=0.6 . Errorbars represent 95% confidence intervals.

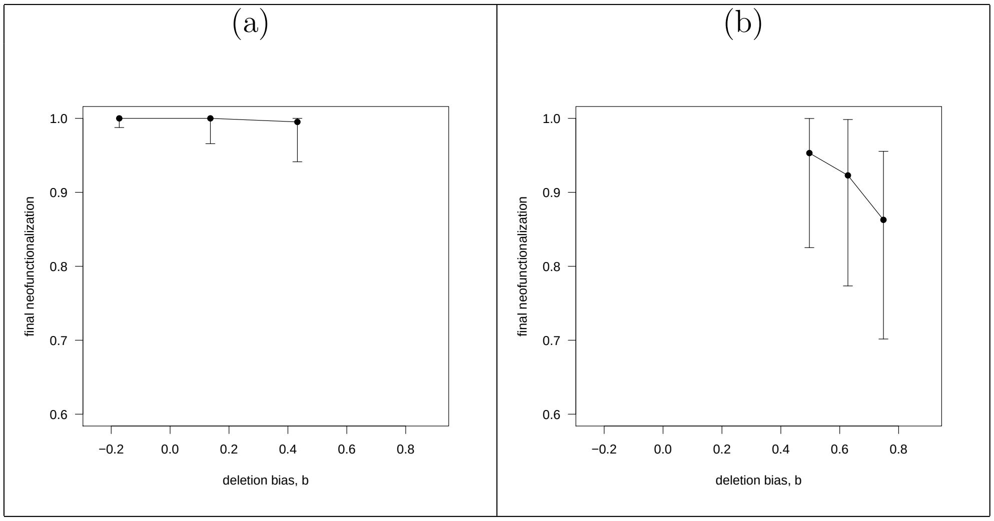


**Supplementary Figure 2.** Final (at time $t = 10000$) neofunctionalization for a range of deletion bias, $b$, values, for both $D = 0$ (a) and $D = -0.5$ (b). The end points of each curve (left end: $c_i = 0.6$, right end: $c_i = 0.3$) are significantly different (Mann-Whitney, $P \sim 0$ for both $D = 0$ and $D = -0.5$). Graphs show median values and 95% confidence interval (errorbars) over 200 independent runs.

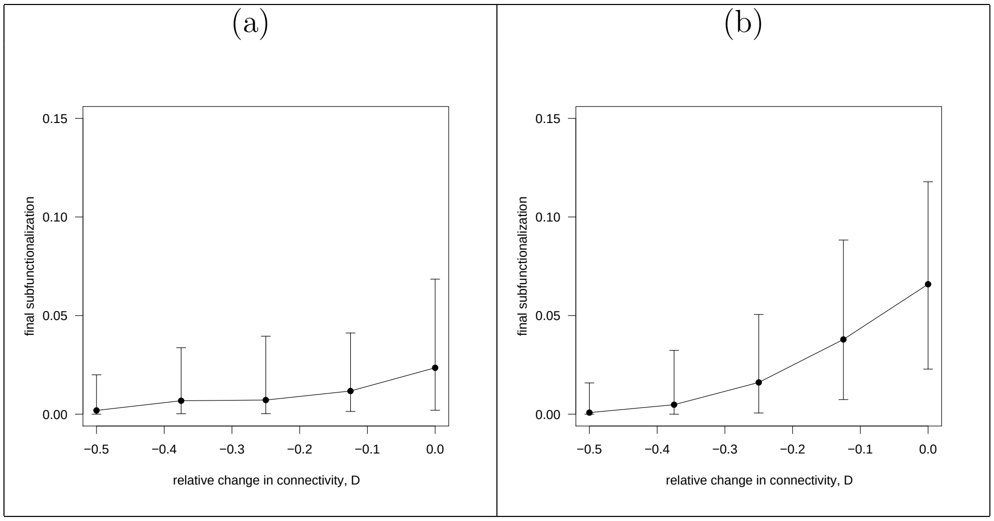


**Supplementary Figure 3.** Subfunctionalization is lower when relative connectivity ($D$) is reduced. Here, we show final (at time $t = 10000$) subfunctionalization across a range of values for $D$, and two values of $c_i$. The end points of each curve ($D = -0.5$ and $D = 0$) are significantly different (Mann-Whitney, $P < 10^{-10}$ for $c_i = 0.3, 0.45, 0.6$). Both graphs show median values and 95% confidence interval (errorbars) over 200 independent runs.

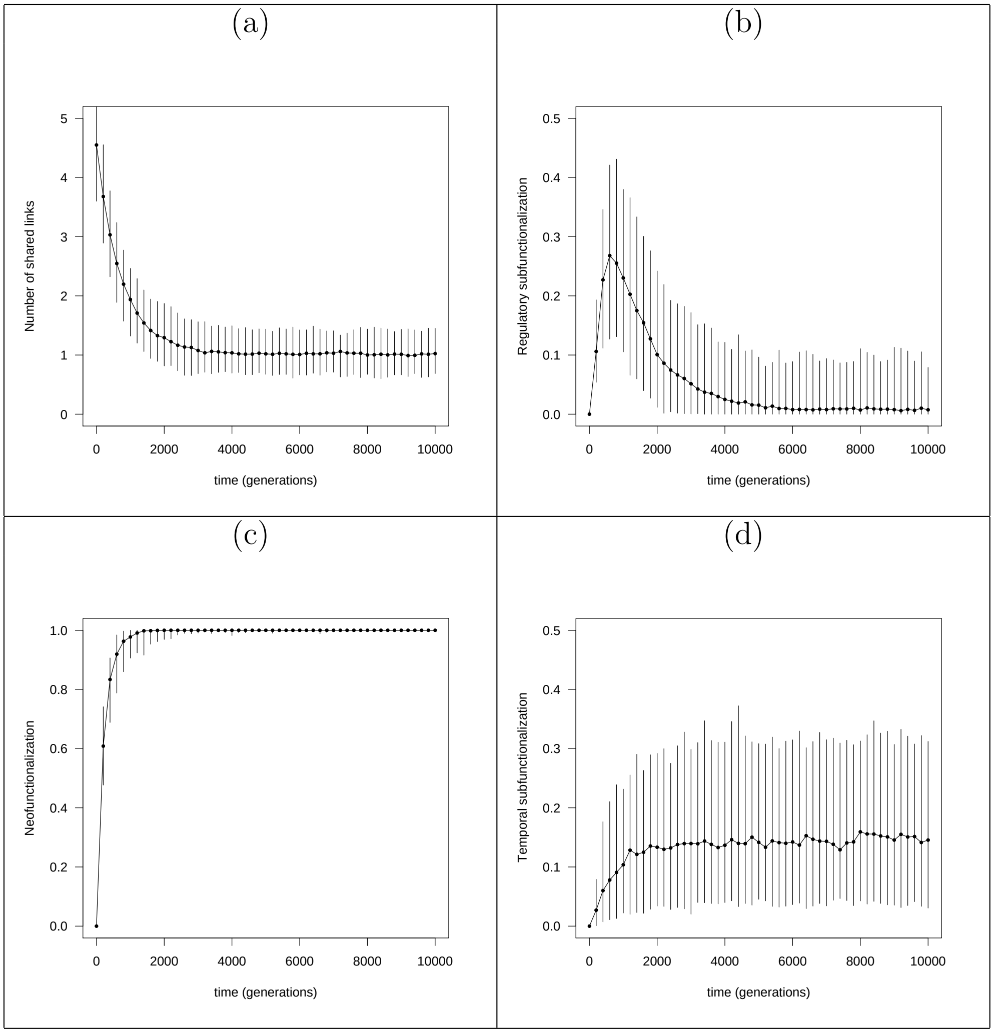


**Supplementary Figure 4.** Unconstrained model, comparable to results for constrained model (figure 3). Evolution of measures over time for a particular set of conditions ($c = 0.45$, $D = 0$). (a) Number of shared regulatory elements ($H$). (b) Regulatory subfunctionalization. (c) Neofunctionalization. (d) Temporal subfunctionalization. Graphs show median values and 95% confidence interval (errorbars) over 200 independent runs.

**Supplementary Table 1.** The results shown in Table 1 are robust to changes in the (arbitrary) parameter $\theta$. The experiments for Table 1 (using $\theta = 0.8$) were repeated for other values of $\theta$ as shown. As in Table 1, we show the frequency of temporal subfunctionalization for the youngest and oldest groups in each dataset, and the computed P-value for the randomized time-courses. P<0.025 indicates the actual frequency is significantly below that expected by chance (see Methods - Analysis of yeast data), and P>0.975 that it is significantly greater. Note that testing $\theta$ <0.5 makes no sense since expression levels could be both ON and OFF, and similarly, testing $\theta$ close to 1 is unreasonable, since it excludes most genes from being ON or OFF.

| | Youngest | | Oldest | |
|---|---|---|---|---|
| Dataset | f(TSF) | P | f(TSF) | P |
| (parameter $\theta$=0.5) | | | | |
| alpha | 0.661 | 0 | 0.830 | 0.014 |
| cdc15 | 0.648 | 0 | 0.907 | 0.199 |
| elutriation | 0.478 | 0 | 0.761 | 0.108 |
| $\alpha$30 | 0.663 | 0 | 0.745 | 0.001 |
| $\alpha$38 | 0.420 | 0 | 0.740 | 0.021 |
| (parameter $\theta$=0.6) | | | | |
| alpha | 0.261 | 0 | 0.631 | 0.117 |
| cdc15 | 0.259 | 0 | 0.630 | 0.037 |
| elutriation | 0.141 | 0 | 0.545 | 0.094 |
| $\alpha$30 | 0.200 | 0 | 0.532 | 0 |
| $\alpha$38 | 0.140 | 0 | 0.410 | 0 |
| (parameter $\theta$=0.7) | | | | |
| alpha | 0.107 | 0 | 0.338 | 0.102 |
| cdc15 | 0.203 | 0.001 | 0.352 | 0.114 |
| elutriation | 0.054 | 0 | 0.293 | 0.065 |
| $\alpha$30 | 0.084 | 0 | 0.277 | 0.002 |
| $\alpha$38 | 0.030 | 0 | 0.240 | 0.002 |
| (parameter $\theta$=0.9) | | | | |
| alpha | 0.000 | 0 | 0.046 | 0.485 |
| cdc15 | 0.000 | 0 | 0.000 | 0.000 |
| elutriation | 0.000 | 0 | 0.076 | 0.633 |
| $\alpha$30 | 0.000 | 0 | 0.213 | 0.081 |
| $\alpha$38 | 0.000 | 0 | 0.030 | 0.026 |

(Results for parameter $\theta$=0.8 are shown in Table 1)
